# Oxidative Stress Biomarkers in Chronic Obstructive Pulmonary Disease Exacerbations: A Systematic Review

**DOI:** 10.3390/antiox10050710

**Published:** 2021-04-29

**Authors:** Elisabetta Zinellu, Angelo Zinellu, Alessandro G. Fois, Maria Carmina Pau, Valentina Scano, Barbara Piras, Ciriaco Carru, Pietro Pirina

**Affiliations:** 1 Clinical and Interventional Pulmonology, University Hospital Sassari (AOU), 07100 Sassari, Italy; elisabetta.zinellu@aousassari.it (E.Z.); agfois@uniss.it (A.G.F.); 2 Department of Biomedical Sciences, University of Sassari, 07100 Sassari, Italy; azinellu@uniss.it (A.Z.); carru@uniss.it (C.C.); 3 Department of Medical, Surgical and Experimental Sciences, University of Sassari, 07100 Sassari, Italy; mcpau@uniss.it (M.C.P.); valescano93@gmail.com (V.S.); barbara.piras@aousassari.it (B.P.)

**Keywords:** COPD, exacerbations, oxidative stress, biomarkers

## Abstract

Background: Chronic Obstructive Pulmonary Disease (COPD) is a progressive disease characterized by a not fully reversible airflow limitation associated with an abnormal inflammatory response. Exacerbations of COPD are of major importance in the acceleration of disease progression, in healthcare costs, and negatively affect the patient’s quality of life. Exacerbations are characterized by a further increase in the airway inflammation likely driven by oxidative stress. In order to deepen the knowledge about this topic, several studies have focused on oxidative stress biomarkers levels. This review summarizes the literature findings about oxidative stress biomarkers in exacerbated COPD patients compared to ones in the stable state. Methods: a systematic search in electronic databases Pubmed, Web of Science, Scopus and Google Scholar from inception to January 2021, was conducted using the terms: “oxidative stress”, “chronic obstructive pulmonary disease” or “COPD”, “exacerbation”. Results: 23 studies were selected for the systematic review. They showed the presence of an imbalance between oxidant and antioxidant molecules in favor of the former in exacerbation of COPD. Conclusions: future studies using standardized methods in better characterized population are needed. However, this review suggests that targeting oxidative stress could be useful in monitoring the disease progression in COPD patients and especially in those more susceptible to exacerbations.

## 1. Introduction

Chronic obstructive pulmonary disease (COPD) is characterized by progressive airflow limitation, not fully reversible, usually caused by exposure to noxious particles or gases [1]. This exposure arouses an abnormal inflammatory response in susceptible persons that causes small airway narrowing and destruction of lung parenchyma with subsequently decline of the lung functions [2]. Cigarette smoking is the most studied among COPD risk factor, though there is evidence that nonsmokers may also develop chronic airflow limitation [3]. This observation supports the role of the individual genetic background in the pathogenesis of COPD [4]. The inflammatory response in patients with COPD involves both innate and adaptive immune system with increased numbers of neutrophils, macrophages, T lymphocytes and B lymphocytes [5]. A key role in driving COPD-related inflammation is played by oxidative stress [2]. This term refers to the state when oxidant activity overwhelms that of antioxidants [6]. Cigarette smoke and environmental pollutants expose the lungs to an excess of oxidants, in particular reactive oxygen and nitrogen species (ROS/RNS). ROS generate airway inflammation leading to the production of proinflammatory cytokines and directly recruiting inflammatory cells which in turn generate ROS, increasing the oxidative stress burden [7]. ROS and RNS are known to damage cellular biomolecules such as lipids, proteins, and polynucleotides. A defense system including non-enzymatic molecules (protein and non-protein thiols and vitamins) as well as enzymatic antioxidants, prevents uncontrolled ROS increase [8]. The imbalance between the production of oxidants and antioxidant defenses may also contribute to the worsening of the disease during acute exacerbations [9]. The acute exacerbations of COPD are characterized by a modification in the patient’s baseline dyspnea, cough, and/or sputum production that may need a change in regular medication [1]. They are triggered predominantly by respiratory viruses and bacteria, which infect the airways and increase airway inflammation [10]. Patients who frequently experience exacerbations have a negative impact on their quality of life and on disease progression. Moreover, exacerbations contribute significantly to healthcare costs of COPD [11]. Therefore, prevention of exacerbations represents a key target for the management of COPD. In order to better characterize the role of oxidative stress in COPD pathophysiology, several studies have investigated oxidative stress biomarkers in stable and exacerbated COPD patients. It is known that in stable COPD compared to control subjects there is an imbalance between oxidant and antioxidant biomarkers in favor of the former [12,13]. This systematic review aims to summarize the literature findings about oxidative stress biomarkers in exacerbated COPD patients compared to the stable state of the disease.

## 2. Search Strategy and Study Selection

**Figure 1:**
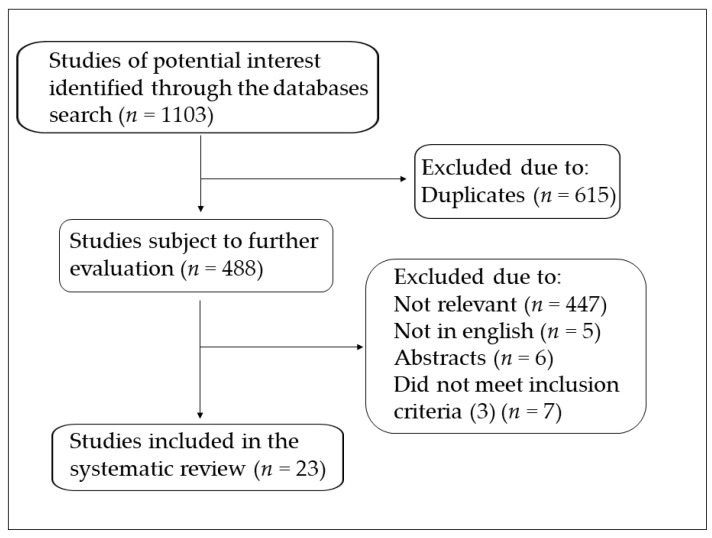
Flow chart of study selection.

A systematic search of publications in electronic databases Pubmed, Web of Science, Scopus, and Google Scholar from inception to January 2021, was conducted using the following keywords: “oxidative stress”, “chronic obstructive pulmonary disease” or “COPD”, “exacerbation”. The criteria used to consider the studies eligible for the review were the following: (1) assessment of oxidative stress biomarkers in any type of biological specimen from COPD patients; (2) comparison of COPD patients in exacerbation and stable state (case-control design); (3) to be considered stable at least one month must have elapsed since the end of the exacerbation, (4) English language; (5) full text available. A flow chart showing the study selection is presented in Figure 1. A total of 23 studies were included in the systematic review.

## 3. Oxidative Stress Biomarkers in Exacerbated COPD 

### 3.1. Brief Overview

The biomarkers that have been evaluated in the included studies are summarized in the Table 1. Both oxidant and antioxidant biomarkers have been considered in several biological sources. The most studied biomarker was malondialdehyde (MDA), a product derived from the oxidation of polyunsaturated fatty acids. It has been evaluated predominantly in blood and to a minor extent in sputum and in exhaled breath condensate (EBC). It has been analyzed mostly as thiobarbituric acid (TBA)-MDA adduct and in one study as free MDA. Another product derived from lipid peroxidation is 8-isoprostane that has been studied in sputum and in EBC. Protein carbonyls, a protein oxidation derived product, have been studied in plasma in one study only. 8-Hydroxy-2′-deoxyguanosine (8-OHdG) is an end-product of oxidative DNA damage that has been studied in plasma in one study. In addition to the byproducts derived from the oxidation of biomolecules, which give an indirect measure of the oxidant status, few studies have measured ROS directly, as hydrogen peroxide (H_2_O_2_) evaluated in EBC in one study, or overall reactive oxygen metabolites (dROMs) and total oxidant status (TOS) measured in serum. Regarding antioxidant markers both enzymatic and non-enzymatic molecules have been considered. Four studies have measured superoxide dismutase (SOD) activity in blood and two in sputum; glutathione peroxidase (GSH-Px) activity has been measured in three studies in plasma and sputum; γ-glutamyltransferase (GGT) activity has been assessed in two studies in serum; catalase has been evaluated in one study in serum and sputum; paraoxonase (PON) and glutathione-S-transferase (α-GST) have been studied in serum and myeloperoxidase (MPO) in sputum in one study. Among non-enzymatic antioxidant molecules, reduced glutathione (GSH) has been evaluated in blood in two studies as well as in sputum and bronchoalveolar lavage fluid (BALF) in one study, while total GSH has been assessed in one study in sputum; protein thiols have been measured in only one study in plasma. Trolox Equivalent Antioxidant Capacity (TEAC) has been assessed in plasma in two studies whilst Total Antioxidant Capacity (TAC) in sputum in one study; vitamins have been evaluated in three studies in serum. The results of these studies are described more fully in the following sections.

### 3.2. Blood Biomarkers

In general, the blood was the most studied as a biological source of oxidative stress biomarkers, as it was seen in stable COPD compared to healthy controls [13]. This may be due to the easier and non-invasive accessibility of blood respect to other biological specimens. Rahman et al. investigated MDA in 11 exacerbated patients compared to 9 patients in the stable state finding a significant increase during the exacerbation [14]. In these patients they also investigated protein carbonyls and protein thiols finding no significant differences and a decrease in the exacerbated group compared to the stable one, respectively. In 20 exacerbated patients and in 29 patients in the stable state, they investigated TEAC finding again a marked reduction in the exacerbated group of patients. Some of the exacerbated patients had been started with corticosteroid therapy during the week before admission. However, the authors found that these patients had plasma TEAC levels similar to those who did not receive corticosteroids. They also found a significative positive correlation between protein thiols and TEAC in exacerbated patients and a negative correlation between superoxide anion released by stimulated neutrophils and plasma antioxidant capacity. Çalikoğlu et al. found increased levels of MDA in 21 patients with exacerbation compared to 41 COPD patients in the stable state [15]. They also found reduced levels of vitamin C and erythrocyte reduced GSH in the exacerbation state. Moreover, they investigated the effect of smoking on this oxidant/antioxidant imbalance, dividing patients on the basis of their smoking status. They observed no differences of these biomarkers’ levels between smokers and non-smokers in both acute exacerbation and stable state of the disease. Tug et al. found increased levels of MDA in 24 exacerbated COPD compared to the same patients evaluated for the stable state 30 days after the end of the exacerbation [16]. They also measured the levels of vitamin C, A, and E. They found not significant differences in Vitamin C concentrations and significant reduction of Vitamin A and E concentrations in exacerbation compared to the stable phase. The same authors measured MDA with two methods (TBARS and HPLC) in 22 male patients at the onset of an exacerbation and then 1 month after the end of the exacerbation [17]. Although they detected different levels of MDA depending on the assay type, a significant increase of MDA in the exacerbation state compared with stable period was observed with both methods. Hanta et al. investigated MDA levels and SOD in 71 patients with stable COPD and 31 patients with exacerbation, finding no differences for both biomarkers [18]. The patients were comparable for sex and age, but not for pack years, statistically higher in the exacerbated patients. When they evaluated the effect of smoking status on plasma MDA levels and erythrocyte SOD activity, they found that the mean values in current smokers were higher, although not statistically significant, than those of the ex-smokers in both groups of patients. Sadowska et al. studied SOD, GSH-Px, and TEAC in 12 exacerbated COPD patients and in 17 stable patients matched for age, gender, and smoking status [19]. They found a significant increase in GSH-Px, a significant reduction in TEAC, and no differences in SOD activity in the exacerbated group compared to the stable group. They also investigated the levels of vitamins E and A, finding significant lowest levels during the exacerbation. Koutsokera et al. explored oxidative stress measuring d-ROM in 30 patients at the onset of their exacerbation and at various times during recovery [20]. They found no differences in oxidative stress during the recovery. The last measurement was taken on the 40th day, about a month after the end of the exacerbation, thus in a stable phase, and still revealed no differences compared to the exacerbation phase. Zeng et al., examined plasma MDA levels in 43 exacerbated patients and in 35 stable patients, matched for age, gender and current smoking [21]. They found increased MDA levels in the exacerbation state. They also studied some antioxidant markers in plasma, such as SOD, reduced GSH, and GSH-Px, finding them reduced in exacerbation in comparison with the stable state. Stanojkovic et al. studied oxidative stress measuring TOS and PON activity in 85 patients at the onset of the exacerbation and about 30 days after, in the stable state of the disease [22]. They found a slight, not significant increase of both parameters in the exacerbation. Ermis et al., investigated the activity of GGT, involved in antioxidant glutathione resynthesis, in 132 exacerbated patients and in 147 stable COPD patients with no differences for gender ratio, age and smoking history [23]. They found a significant increase of this enzyme activity in the exacerbated group compared to the stable one. They also found a significant positive correlation between the GGT activity and C reactive protein (CRP) measurements. Antus et al., explored the activity of SOD and catalase in 36 exacerbated patients and in 24 stable COPD patients [24]. They found a not significant reduction of these parameters in the exacerbated group serum. Liu et al. investigated the plasma levels of 8-OHdG in 110 COPD patients with exacerbation and in 24 COPD patients in a stable state [25]. The patients were comparable for age and gender while, regarding the smoking status, the stable COPD had a percentage of current smokers higher than the exacerbated COPD. The authors found significantly higher levels of 8-OHdG in exacerbated patients. They also found that 8-OHdG level was positively associated with symptoms severity, with some inflammatory markers such as CRP and negatively associated with lung function. Sun et al., explored serum levels of GGT in 117 COPD patients with exacerbation and in 107 stable COPD patients comparable for age, gender, and smoking status [26]. They found increased levels of GGT in the exacerbated group. They also found significant negative correlations between GGT levels and lung function. Yin et al., measured serum α-GST in 40 AECOPD patients and in 10 stable COPD, finding no differences between the two groups [27]. 

### 3.3. Sputum Biomarkers 

The sputum has been used in several studies to investigate local oxidative stress in COPD. This method can reflect the composition of airway secretions in a not invasive way. Some authors have performed their studies using spontaneously expectorated sputum in exacerbated patients and induced sputum in patients in the stable state. In these individual studies the authors themselves clarified that the induction of sputum did not affect the measure of the studied biomarkers respect to spontaneously collected sputum. Kersul et al., investigated total antioxidant status in induced sputum in 17 patients at the onset of an exacerbation and three month later in a stable state finding a significant reduction of this biomarker in the exacerbation [28]. Zeng et al., investigated oxidant and antioxidant biomarkers also in induced sputum [21]. They found increased levels of MDA, decreased levels of reduced glutathione, and diminished SOD and GSH-Px activity in exacerbation compared to stable patient as they detected in plasma. They also found a positive correlation between plasma levels of MDA, reduced glutathione, SOD and GSH-Px activity and the same biomarkers levels in induced sputum. Tufvesson et al. investigated 8-isoprostane and MPO first in 43 COPD patients in their stable state and then in 25 of these patients that had an exacerbation within six months [29]. They observed an increase of both biomarkers in induced sputum in the exacerbation phase, although not significant. Turgut et al., examined total glutathione in induced sputum of 11 exacerbated COPD and 10 stable COPD finding a not significant increase in the exacerbated group [30]. Antus et al. explored MDA levels in spontaneously expectorated sputum of 34 exacerbated COPD and in induced sputum of 21 stable patients comparable for smoking history, age, and gender [31]. They found a significant increase of this marker in the exacerbated group. Drozdovszky et al., investigated another lipid peroxidation derived product, 8-isoprostane, in spontaneously expectorated sputum of 25 AECOPD and in induced sputum of 37 stable COPD [32]. The two groups of patients were comparable for smoking history, age, and gender. The authors found increased levels of 8-isoprostane in the exacerbated group. Antus et al., examined MDA, SOD, and catalase activity in expectorated sputum of 36 exacerbated COPD patients and in induced sputum of 24 clinically stable COPD patients [24]. They found that MDA levels and antioxidant enzymes activity were significantly increased in exacerbated patients compared to stable patients. They also found significant positive correlations in sputum between SOD or catalase activities and the number of neutrophils in both stable and AECOPD groups. Moreover, sputum MDA levels correlated positively with SOD and CAT activities in AECOPD. 

### 3.4. EBC and BALF Biomarkers

EBC sampling is a noninvasive, rapid, and easily repeatable method suitable to the analysis of COPD biomarkers. Dekhuijzen et al. found a significant increase of H_2_O_2_ in EBC of 19 exacerbated COPD and of 12 stable COPD [33]. Biernacki et al. measured 8-isoprostane in EBC of 21 exacerbated COPD patients and in 12 of them two months after the exacerbation when they were in the stable phase [34]. A significant reduction of 8-isoprostane was observed in the 12 stable patients. They also found no significant correlation between the degree of airways obstruction and the levels of 8-isoprostane in exacerbated COPD. Carpagnano et al., studied 8-isoprostane in EBC of 40 ex-smoker COPD patients, 30 of which were exacerbated and the remaining 10 were stable [35]. They observed significant higher levels of this lipid peroxidation biomarker in the exacerbated group compared to the stable one. Antus et al., investigated MDA also in EBC as well as in sputum [31]. They found similar levels of MDA between exacerbated and stable patients and found no correlations between EBC MDA values and lung function. Only one study investigated BALF. Drost et al., investigated glutathione in the BAL of 7 patients with stable COPD and in 14 exacerbated patients divided on the basis of exacerbation severity: those with severe exacerbations requiring hospital admission and those with very severe exacerbations who had respiratory failure and required intervention with mechanical ventilation [36]. They found a reduction of glutathione in very severe exacerbated compared to stable COPD and a further reduction in severe exacerbation.

## 4. Discussion

The COPD is the most prevalent and the first leading cause of death among all chronic respiratory diseases [37]. The progression of COPD is variable; in some patients the disease has a rather stable course while others experience exacerbations that play a major role in the natural history of COPD and are associated with significant morbidity, mortality, and economic burden. Exacerbations are more frequent and severe as the severity of the underlying COPD increases, although an independent susceptibility phenotype appears to exist [38]. A better understanding of the mechanisms of COPD exacerbations is needed in order to improve their management and therapeutic strategies. Respiratory viral and bacterial infections are the main cause of COPD exacerbations, although environmental factors can worsen these events [10]. There is a consequent increase of airway inflammation that likely involves oxidative stress which induces proinflammatory cytokines and chemokines production, reduces the activity of antiproteases and the expression of anti-inflammatory genes such as histone deacetylase-2 [39]. Moreover, inflammatory cells such as activated neutrophils and macrophages, as well as lung epithelial cells, increase further the oxidative burden representing an important endogenous source of reactive oxygen species [7]. Given the known role that oxidative stress plays in the pathogenesis of COPD and in its progression, it is necessary to deepen the knowledge on this topic. The extent of oxidative stress can be estimated by measuring several biomarkers. In this regard this review summarizes the literature findings about oxidative stress biomarkers in exacerbated COPD patients compared to the stable phase of the disease. In some studies, different groups of patients were compared, in others the same group of exacerbated patients was evaluated again in the stable phase. In this last case, to avoid the possible influence of drugs used to treat the exacerbation, only studies in which at least one month had elapsed since the end of the exacerbation were selected. It emerges that lipid peroxidation products are the most studied among oxidative stress biomarkers, in particular MDA that has been described increased in the majority of studies performed in blood, in all the studies performed in sputum, while the only study that investigated MDA in EBC found no differences between exacerbated and stable COPD. 8-isoprostane was found increased in all the studies carried out in sputum and EBC. Therefore, it seems that MDA and 8-isoprostane represent reliable markers to evaluate the degree of oxidative stress. Protein carbonyls TOS, 8-OHdG, d-ROMs, and H_2_O_2_ have been evaluated in exacerbations compared to stable state, each of them in only one study. Hence, more studies should be carried out to confirm the observed results. Regarding antioxidant markers, they have been studied in few studies and in different biological specimens giving results not always concordant. The antioxidant capacity evaluated by TEAC or TAC has been found reduced in exacerbated COPD compared to the stable state. Additionally, reduced glutathione, assessed in four studies in blood, sputum and BALF has been found reduced in exacerbation. Conversely, other biomarkers such as vitamins and antioxidant enzymes activity showed conflicting results as it was observed also in stable COPD compared to healthy subjects [40]. It is likely that these molecules can be mostly affected by individual factors such as the kind of diet, the physical activity and the variables related to lifestyle that are not considered in the single studies. The lack of a standardized characterization of subjects represents a limitation of this systematic review, as well as the relatively low number of subjects evaluated in single studies. On the other hand, highlighting these limitations it also underlines the need to improve the research in this field involving a greater number of better-defined subjects. In this regard, it would be interesting to better characterize COPD patients on the basis of exacerbations frequency compared to patients with a more stable course of disease. As far as we know this is the first systematic review about oxidative stress biomarkers in exacerbated COPD compared to the stable state. It shows the presence of an imbalance between oxidant and antioxidant molecules in favor of the former and suggests that targeting oxidative stress could be useful in monitoring disease progression in COPD patients and especially in those more susceptible to exacerbations. Several approaches have been investigated to reduce oxidative stress in animal models of COPD, some of which have been tested clinically [41]. Thiol-based molecules, such as N-acetylcysteine, erdostein, and carbocysteine, have been the most studied in clinical studies and appear to have positive effects on the rate and duration of exacerbations [42,43,44]. Therefore, it is reasonable to assume that targeting oxidative stress represents a strategy that deserves to be further investigated. In order to pursue this goal, a better knowledge of oxidative stress status in COPD exacerbations is desirable. In this context the present review provides a solid tool for future studies.

## Figures and Tables

**Table 1:** Oxidative stress biomarkers in exacerbated COPD patients (AE) compared to stable (ST).

Oxidative Stress Biomarkers	Biological Specimen	AE (n)	ST (n)	Ref
MDA ↑; protein carbonyls ↔; TEAC ↓; protein thiols ↓	plasma	11	9	[14]
MDA ↑; vitamin C ↓	serum			
reduced GSH ↓	erythrocytes	21	41	[15]
MDA ↑; vitamin C ↔; vitamin A and E ↓	serum	24	24	[16]
MDA ↑;	serum	22	22	[17]
MDA ↔	plasma			
SOD ↔	erythrocytes	31	71	[18]
TEAC ↓; Vitamin A and E ↓	plasma			
GSH-Px ↑; SOD ↔	whole blood	12	17	[19]
dROM ↔	serum	30	30	[20]
MDA ↑; SOD ↓; GSH-Px ↓; reduced GSH ↓	plasma			
MDA ↑; SOD ↓; GSH-Px ↓; reduced GSH ↓	sputum	43	35	[21]
TOS ↑ns; PON↑ns	serum	85	85	[22]
GGT ↑	serum	132	147	[23]
SOD ↓ns; catalase ↓ns	serum			
MDA ↑; SOD ↑; catalase ↑	sputum	36	24	[24]
8-OHdG ↑	plasma	110	24	[25]
GGT ↑	serum	117	107	[26]
GST ↔	serum	40	10	[27]
TAC ↓	sputum	17	17	[28]
8-isoprostane ↑ ns; MPO ↑ ns	sputum	25	43	[29]
total GSH ↑ ns	sputum	11	10	[30]
MDA ↑	Sputum			
MDA ↔	EBC	34	21	[31]
8-isoprostane	sputum	25	37	[32]
H2O2 ↑	EBC	19	12	[33]
8-isoprostane ↑	EBC	21	12	[34]
8-isoprostane ↑	EBC	30	10	[35]
reduced GSH ↓	BALF	14	7	[36]

↑ increased levels; ↓ reduced levels; ↔ no differences; ns: not significant. GGT: γ-glutamyltransferase; GPx: glutathione peroxidase; GSH: glutathione; GST: Glutathione-S-transferase; H_2_O_2_: hydrogen peroxide; MDA: malondialdehyde; MPO: myeloperoxidase; PON: paraoxonase; dROM: reactive oxygen metabolites; SOD: superoxide dismutase; TAC: Total Antioxidant Capacity; TEAC: Trolox Equivalent Antioxidant Capacity; TOS: Total Oxidative Status; 8-OHdG: 8-hydroxy-2′-deoxyguanosine.

## Data Availability

Data is contained within the article.
